# Differential levels of anti-*Mycobacterium tuberculosis*-specific IgAs in saliva of household contacts with latent tuberculosis infection

**DOI:** 10.3389/fmed.2023.1267670

**Published:** 2023-10-06

**Authors:** Cinthya Ruiz-Tagle, Rodrigo Naves, Patricia García, Anna Günther, Nicole Schneiderhan-Marra, María Elvira Balcells

**Affiliations:** ^1^Departamento de Enfermedades Infecciosas del Adulto, Escuela de Medicina, Pontificia Universidad Católica de Chile, Santiago, Chile; ^2^Instituto de Ciencias Biomédicas, Facultad de Medicina, Universidad de Chile, Santiago, Chile; ^3^Laboratorio de Microbiología, Departamento de Laboratorios Clínicos, Escuela de Medicina, Pontificia Universidad Católica de Chile, Santiago, Chile; ^4^NMI Natural and Medical Sciences Institute at the University of Tübingen, Reutlingen, Germany

**Keywords:** tuberculosis, saliva, immunoglobulin, IgA, antibody, biomarker, diagnostic test, *Mycobacterium tuberculosis*

## Abstract

**Introduction:**

Mucosal immunity is strongly elicited in early stages of many respiratory and enteric infections; however, its role in tuberculosis pathogenesis has been scarcely explored. We aimed to investigate *Mycobacterium tuberculosis* (Mtb) specific IgA levels in saliva in different stages of latent Tuberculosis Infection (TBI).

**Methodology:**

A multiplex bead-based Luminex immunoassay was developed to detect specific IgA against 12 highly immunogenic Mtb antigens. A prospective cohort of household contacts (>14 years) of pulmonary TB cases was established in Santiago, Chile. Contacts were classified as Mtb-infected or not depending on serial interferon-γ release assay results. Saliva samples were collected and tested at baseline and at a 12-week follow-up.

**Results:**

Mtb-specific IgA was detectable at all visits in all participants (*n* = 168), including the “non-Mtb infected” (*n* = 64). Significantly higher median levels of IgA were found in the “Mtb infected” compared to the uninfected for anti-lipoarabinomannan (LAM) (110 vs. 84.8 arbitrary units (AU), *p* < 0.001), anti-PstS1 (117 vs. 83 AU, *p* < 0.001), anti-Cell Membrane Fraction (CMF) (140 vs. 103 AU, *p* < 0.001) and anti-Culture Filtrate Proteins (CFP) (median 125 vs. 96 AU, *p* < 0.001), respectively. Nonetheless, the discriminatory performance of these specific mucosal IgA for TBI diagnosis was low.

**Conclusion:**

Saliva holds Mtb-specific IgA against several antigens with increased levels for anti-LAM, anti-PstS1, anti-CMF and anti-CFP found in household contacts with an established TBI. The role of these mucosal antibodies in TB pathogenesis, and their kinetics in different stages of Mtb infection merits further exploring.

## 1. Introduction

Tuberculosis (TB) is an airborne disease caused by *Mycobacterium tuberculosis* (Mtb) infection and is the leading cause of death among infectious diseases from a single agent, with over 10 million people developing TB and 1.2 million deaths annually (1). Tuberculin skin test (TST) and interferon-γ release assay (IGRA) are immune-based tests used to detect Mtb infection after exposure. Despite having good sensitivity and specificity, these tests do not distinguish between latent TB infection (TBI) and TB disease, are not able to predict risk of progression from TBI to TB disease, nor can they differentiate recent infection from long-time established TBI. Additionally, they may fail to detect recently acquired TBI (2–7). Currently, there is no gold standard for the diagnosis of TBI and therefore new research strategies are being developed for the diagnosis of TBI (8).

The protective role of humoral immunity in TB pathogenesis has largely been disregarded, as cellular defense mechanisms are more important due to the intracellular nature of Mtb (9–11). Notwithstanding, increasing evidence has demonstrated that B cells and antibodies can also contribute to immunity against Mtb (12–14). Mouse models have shown that anti-arabinomannan IgG (15), anti-lipoarabinomannan (LAM) IgG (12), anti-HspX IgA (16) and anti-heparin-binding hemagglutinin adhesin (HBHA) (17) are able to confer partial protection after a respiratory challenge with Mtb. Antibody-mediated protection has also been observed in humans both *in vivo* and *in vitro*. Children who do not develop antibody response or exhibit low levels of serum IgG against lysates of slow-growing mycobacteria and purified LAM, are more prone to TB dissemination (18). *In vitro*, anti-arabinomannan IgG in serum, and its oligosaccharide fragments, facilitate opsonization, favoring phagolysosome fusion and reduction of *Mycobacterium bovis* BCG (BCG) intracellular growth in human macrophages (19). A recent comprehensive literature about the role of antibodies in TB pathogenesis can be found in the review by Melkie et al. (20).

Humoral immune response in mucosae is mainly mediated by secretory IgA, which is considered its hallmark antibody. IgA is resistant to proteases and functions by neutralizing pathogens, toxins, and allergens, as well as mediating an anti-inflammatory response (21). Salivary IgA is produced in salivary glands by local plasma cells, including those activated in the nasopharynx-associated lymphoid tissue (NALT) (22) which stands in the front line defense for airborne pathogens. During human exposure, Mtb reaches the lung through the upper airways passages, and transient Mtb detection has been reported in the upper respiratory tract mucosa of household contacts of pulmonary TB (PTB) cases (23, 24). Mtb does not usually penetrate upper respiratory mucosa, but translocation across M-cells in NALT has been shown to occur both *in vitro* and in mice models (25). Although there are limited studies addressing the protective role of mucosal immune response in mycobacterial infections, increased susceptibility to intranasal infection with BCG has been shown in IgA and IgA receptor deficient mice (26, 27). Accordingly, higher levels of salivary anti-PstS1 IgA have been described in a population of Warao Amerindian children with TBI compared to uninfected children (28, 29), as was the case in saliva from patients with PTB compared to uninfected controls (30).

In this study, we developed an immunoassay to detect Mtb-specific IgA antibodies in saliva, and explored its secretion in household contacts after TB disease exposure, to generate information about the role of the mucosal humoral immunity in TB pathogenesis and search for potential new biomarkers for early stages of TBI.

## 2. Materials and methods

### 2.1. Patient selection

**Figure 1:**
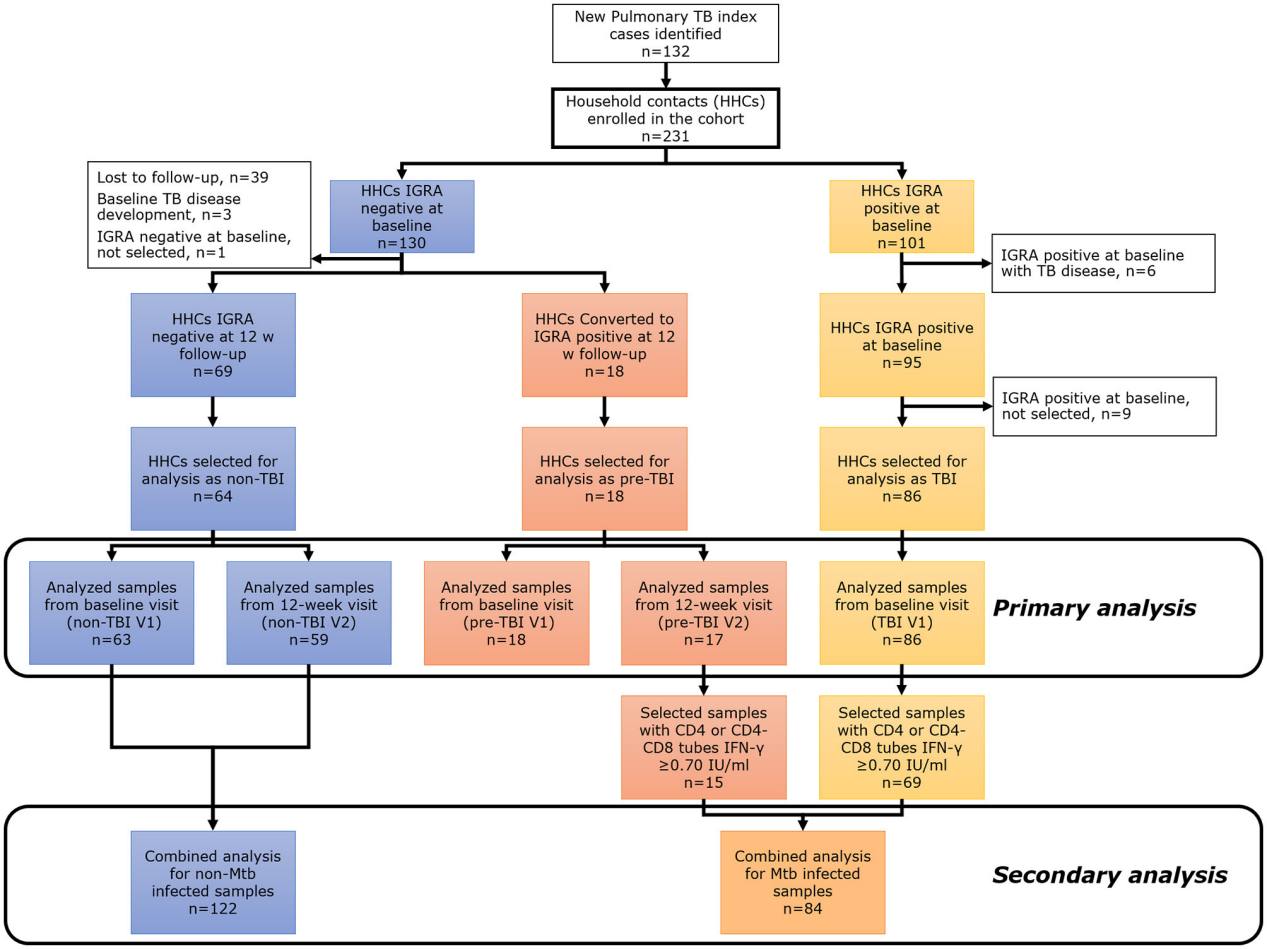
Main study flowchart for primary and secondary analysis. The primary analysis compared “non-TBI”, “pre-TBI”, and “TBI” groups at baseline (V1) and follow-up (V2). The secondary analysis compared the “non-Mtb infected” and “Mtb infected” groups.

A prospective cohort of household contacts (>14 years old) of TB cases (only acid-fast smear-positive index cases were included) was conducted between September 2017 and February 2020 in Santiago Metropolitan area, Chile. Saliva samples were collected from all participants and TBI status was assessed by IGRA (QuantiFERON^®^-TB Gold Plus (QFT), QIAGEN, Hilden, Germany). All the contacts were tested with IGRA at a baseline visit (V1) and again at a 12-week visit follow-up (V2) if IGRA result was negative at V1. Chest X-rays and TB symptoms screening were done at baseline, 12 weeks and 24 weeks; then bi-monthly telephone follow-up was completed for up to 1 year. Contacts with known prior history of TB, autoimmune diseases, pregnancy, known HIV infection, current use of corticosteroids or other immunosuppressants, and inhalation drugs were excluded. Final contact classification was as follows: (1) “non-TBI” group: household contacts with a negative IGRA test result at baseline (V1) and at 12-week follow-up (V2); (2) “pre-TBI” group: household contacts with a negative IGRA result at V1 but positive IGRA result at 12-week follow-up (V2); (3) “TBI” group: household contacts with a positive IGRA test result (CD4 or CD8 IFN-γ levels ≥0.35 IU/ml) at V1 (Figure 1). Individuals from the “non-TBI” and “TBI” groups that developed TB disease at V1 or before V2 were excluded.

In a secondary analysis, saliva samples from “non-TBI”, “pre-TBI” and “TBI” groups were classified binarily as: (1) “non-Mtb infected” group: all samples from participants of the “non-TBI” V1 and “non-TBI” V2 groups. (2) “Mtb infected” group: all samples from individuals of the “pre-TBI” V2 and “TBI” V1 groups with a strong IGRA positive result (CD4 or CD8 IFN-γ levels ≥0.70 IU/ml) (Figure 1).

### 2.2. Multiplex bead-based Luminex immunoassay development for mucosal IgA detection against specific Mtb antigens

*Immunoassay*: A multiplex bead-based Luminex immunoassay was developed to detect mucosal IgA antibodies against specific Mtb antigens. We selected twelve Mtb antigens that have previously been reported as highly immunogenic and whose expression have been shown to be present in early stages of the disease (4 to 12 weeks) both in plasma and serum samples from non-human primates Mtb-infected models as well as samples from patients with TB disease (31–38). The twelve antigens were obtained as fragment preparations or native and recombinant proteins, and corresponded to Whole Cell Lysate (WCL), PstS1, ESAT-6, Culture Filtrate Proteins (CFP), Cytosol Fraction (CF), Cell Membrane Fraction (CMF), MPT32, HspX, Antigen 85 Complex A (Ag85A), Antigen 85 Complex B (Ag85B), EsxB (CFP-10) and LAM. The assay was set up according to a previous publication (38) with some modifications. Assay development and validation process was performed with saliva samples from non-TB exposed controls with negative IGRA, and from patients with PTB disease under TB treatment. We selected a few samples to assess the optimal buffer combination, and sample and secondary antibody dilution. We also determined the intra and inter-assay coefficient variations, and sample stability (Supplementary Appendix 1).

*Antigens*: BEI Resources, NIAID, NIH provided the following antigens: Whole Cell Lysate, NR-14822; PstS1 (Gene Rv0934, Non-Acylated), Purified Native Protein from *Mycobacterium tuberculosis*, Strain H37Rv, NR-14859; ESAT-6, Recombinant Protein Reference Standard, NR-49424; Culture Filtrate Proteins, NR-14825; Cytosol Fraction, NR-14834, and *Mycobacterium tuberculosis*, Strain H37Rv, Cell Membrane Fraction, NR-14831. Recombinant antigens MPT32, HspX, Antigen 85 Complex A (Ag85A), Antigen 85 Complex B (Ag85B) and EsxB (CFP-10) were purchased from the Foundation for Innovative New Diagnostics (FIND). Lipoarabinomannan (LAM) from *Mycobacterium tuberculosis* Aoyama-B (#02449-61) was purchased from Nacalai Tesque, Inc.

*Antibodies*: Monomeric human IgA, human IgA lambda (dimer), goat anti-human IgA and goat anti-human IgA-RPE were purchased from Abcam (ab91025, Cambridge, United Kingdom), Gentaur GmbH (P 444, Aachen, Germany) and Jackson ImmunoResearch Inc. (109-005-011 and 109-115-011, Pennsylvania, United States), respectively.

*Reagents*: Magnetic beads, Low Cross Buffer and normal goat serum were purchased from Luminex Corporation (Texas, United States), Candor (Wangen, Germany) and Thermofisher (Massachusetts, United States), respectively.

*Salivary IgA detection*: Frozen saliva samples were thawed, centrifuged at 10, 000 × g for 5 min at room temperature (RT), diluted (1:4) in assay buffer (100 mM NaCl, 20 mM Tris pH 7.0, 50 mM CaCl_2_, 1% Triton X-100) and incubated for 20 min on a shaker. Then, 50 μl of diluted saliva sample was mixed with master bead mix containing antigen coupled microspheres (for each antigen at least 1000 beads per well), and incubated for 2 h at RT with stirring and protected from light. Unbound antibodies were removed by washing the beads five times with 100 μl of washing buffer I (PBS + 1% Tween20). To visualize antigen-bound human IgA, the beads were incubated with 50 μl of an R-PE labeled goat anti-human IgA antibody (7.5 μg/ml) diluted in detector buffer (PBS + Low Cross Buffer + 5 g/L BSA + 2.5% normal goat serum) for 45 min at RT with agitation and protected from light. After washing three times with 100 μl of washing buffer II (PBS + 0.05% Tween20), the beads were resuspended in 80 μl of washing buffer II. Readout was performed using a Luminex MAGPIX instrument (Luminex Corp, Austin, Texas, USA). Binding events were displayed as MFI based on >35 measured beads per bead sort. Assays were performed in duplicate. Valid MFIs were used for data analysis.

### 2.3. Sampling procedures

*Saliva:* Participants were instructed to pool the saliva inside their closed mouth for as long as they could and to deposit it inside a sterile container. The process, repeated until they collected about 1–4 ml, took approximately 20 min depending on the participant. After collection, samples were kept on ice until centrifuged at 10, 000 × g for 15 min at 4°C, aliquoted and stored at −80°C until measurements.

*Blood:* Blood samples were drawn from participants to assess TBI status with a standard commercial IGRA test [QuantiFERON^®^-TB Gold Plus (QFT), QIAGEN, Hilden, Germany].

### 2.4. Ethical approval

Ethical approval was obtained from the Institutional Review Board from the Pontificia Universidad Católica de Chile. All eligible participants provided written informed consent, according to institutional requirements. In the case of minors, written consent was given by a parent. All participants having a positive IGRA result were referred to the local TB program provider to evaluate the need of TBI chemoprophylaxis.

### 2.5. Statistical analysis

Analyses were performed using RStudio (version 1.3.1073) (39). Comparisons between two independent groups were performed using the Mann–Whitney *U*-test. Comparisons for more than two independent groups were performed with the Kruskal–Wallis test. When significant, two groups comparison were performed with Mann–Whitney *U*-test with Bonferroni correction. A receiver operating characteristic curve (ROC) to assess the area under the curve (AUC) was performed only for the secondary analysis (“non-Mtb infected” vs. “Mtb infected” group). For all analyses, 2-sided *p*-values <0.05 were considered statistically significant. For groups comparisons analysis, we only selected samples that met the following criteria of Luminex immunoassay readings: >35 measured beads per bead sort (40), and a CV <20% for the median fluorescence intensity (MFI), measured in arbitrary units (AU). Samples from 12 participants (“non-TBI,” *n* = 6; “pre-TBI,” *n* = 1; “TBI,” *n* = 5) did not meet these criteria and were therefore excluded from the analysis (12 out of 243 samples).

## 3. Results

### 3.1. Assay development measurements of specific salivary IgA levels in non-TB exposed controls and patients with PTB disease

For assay development, we evaluated saliva samples from 27 “non-TB exposed” controls (healthy volunteers with a median age of 42 years (18–59), 51.9% female and 88.9% being from Chilean nationality) and from 17 patients with PTB disease [median age of 29 years (18–52), with 70.6% male and 88.2% from other countries from Latin America].

**Figure 2:**
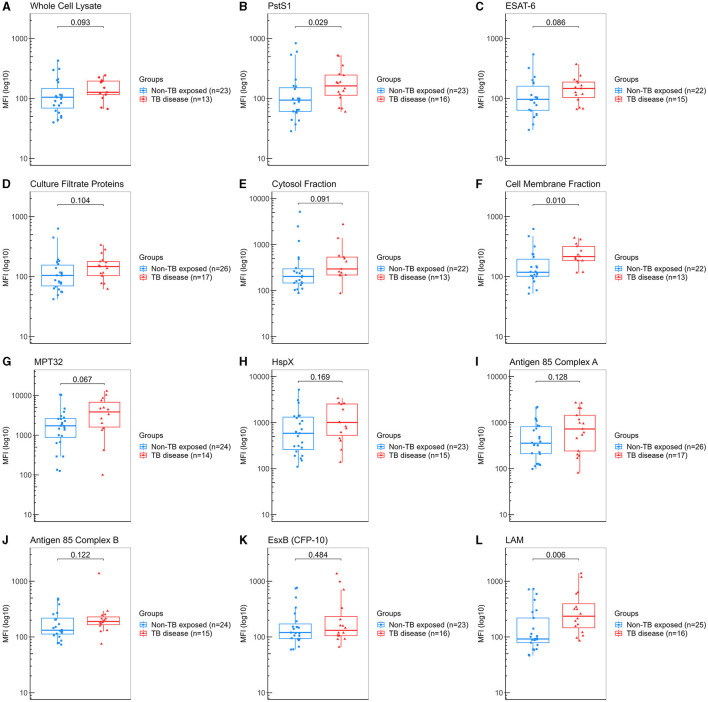
Measurement of IgA against Mtb antigens in saliva samples from “non-TB exposed” controls and patients with TB disease (assay development). Samples were incubated with bead-coupled antigens and IgA bound to bead coupled antigens was recognized with an anti-hu-IgA-R-PE secondary antibody and detected with a MAGPIX instrument (Luminex). Binding events are displayed as median fluorescence intensity (MFI) in arbitrary units (AU) and plotted in a log scale. In the box and whisker plot are represented the median with the lower and upper quantiles, and the minimum and maximum value of the data set for each group. Mtb antigens specific IgAs were as follows: **(A)** anti-whole cell lysate IgA, **(B)** anti-PstS1 IgA, **(C)** anti-ESAT-6 IgA, **(D)** anti-culture filtrate proteins IgA, **(E)** anti-cytosol fraction IgA, **(F)** anti-cell membrane fraction IgA, **(G)** anti-MPT32 IgA, **(H)** anti-HspX IgA, **(I)** anti-Ag85A IgA, **(J)** anti-Ag85B IgA, **(K)** anti-EsxB (CFP-10) IgA and **(L)** anti-LAM IgA. Comparison groups were as follows: (1) “non-TB exposed” controls (*n* = 27) vs. (2) patients with TB disease (*n* = 17). The analysis performed included only samples with ≥35 measured beads per sort; thus, each plot displays the actual number of samples included in the analysis for each antigen IgA. The statistical significance was calculated using the Mann–Whitney *U*-test, and two-tailed *p*-values are indicated.

We determined the IgA levels against the twelve different Mtb-specific antigens by multiplex bead-based Luminex immunoassay. We found that patients with PTB disease showed significantly higher levels for anti-LAM (median 237 vs. 92 AU, *p* = 0.006), followed by CMF (median 216 vs. 118 AU, *p* = 0.010) and PstS1 (median 163 vs. 94 AU, *p* = 0.029) compared to “non-TB exposed” controls (Figure 2). Based on these results, we determined that our assay could detect specific IgA against some of Mtb antigens and thus proceed with evaluating the HHCs cohort.

### 3.2. Cohort study participants

Participants from the household contacts cohort were enrolled between September 2017 and February 2020. The clinical, demographic and epidemiological characteristics of all household contacts included in the study (*n* = 168), primarily categorized as “non-TBI” (*n* = 64), “pre-TBI” (*n* = 18) and “TBI” groups (*n* = 86) are summarized in Table 1. Participants had a median age of 32 years (15–76), with 56.5% female. Most participants (82.9%) had been vaccinated with universal BCG at birth. Over half of contacts (51.2%) had a positive IGRA at baseline. Among those having a negative IGRA at baseline (*n* = 82), 78% remained IGRA negative at follow-up and 22% acquired a TBI (*n* = 18). Among them only 3 developed TB disease after the 12-week follow-up. The characteristics of household contacts whose samples were categorized for the secondary analysis as “non-Mtb infected” and “Mtb infected” groups (*n* = 148) are summarized in Table 2.

**Table 1:** Clinical and epidemiological characterization of household contacts according to group classification: “non-TBI”, “pre-TBI”, and “TBI”.

	**“Non-TBI” (*N =* 64)**	**“Pre-TBI” (*N =* 18)**	**“TBI” (*N =* 86)**	**All household contacts (*N =* 168)**	***p*-value**
**Sex**					
Female	33 (51.6%)	11 (61.1%)	51 (59.3%)	95 (56.5%)	0.578
**Age (years)**					
Median [Min, Max]	32.5 [15.0, 65.0]	24.0 [16.0, 50.0]	33.0 [15.0, 76.0]	32.0 [15.0, 76.0]	0.004
**Race**					
Afro-Caribbean	2 (3.1%)	1 (5.6%)	4 (4.65%)	7 (4.2%)	0.948
Asian	1 (1.6%)	0 (0%)	1 (1.16%)	2 (1.2%)	
Hispanic/Latino	60 (95.3%)	17 (94.4%)	81 (94.19%)	159 (94.6%)	
**Country of origin**					
Peru	25 (39.1%)	10 (55.6%)	47 (54.7%)	82 (48.8%)	0.066
Chile	24 (37.5%)	1 (5.6%)	21 (24.4%)	46 (27.4%)	
Venezuela	7 (10.9%)	1 (5.6%)	4 (4.7%)	12 (7.1%)	
Bolivia	1 (1.6%)	3 (16.7%)	4 (4.7%)	8 (4.8%)	
Colombia	3 (4.7%)	1 (5.6%)	3 (3.5%)	7 (4.2%)	
Haiti	1 (1.6%)	1 (5.6%)	4 (4.7%)	6 (3.6%)	
Other	3 (4.7%)	1 (5.6%)	3 (3.5%)	7 (4.2%)	
**TB disease**					
After 12-week follow-up		3 (16.7%)	1 (1.2%)	4 (2.4%)	
**BCG vaccination** ^†^					
Yes	53 (88.3%)	15 (83.3%)	68 (79.1%)	136 (82.9%)	0.361

TB: Tuberculosis. ^†^BCG vaccine status defined positive if BCG skin mark was present. Information was unavailable for eight participants. “Non-TBI”: household contacts with a negative IGRA test result at baseline and at 12-week follow-up. “Pre-TBI”: household contacts with a negative IGRA result at baseline but positive IGRA result at 12 week-follow-up. “TBI”: household contacts with TBI at baseline by a positive IGRA test result.

**Table 2:** Clinical and epidemiological characterization of household contacts samples according to secondary group classification: “non-Mtb infected” and “Mtb infected”.

	**“Non-Mtb infected” (*N =* 64)**	**“Mtb infected” (*N =* 84)**	**All household contacts (*N =* 148)**	***p-*value**
**Sex**				
Female	33 (51.6%)	53 (63.1%)	86 (58.1%)	0.215
**Age (years)**				
Median [Min, Max]	32.5 [15.0, 65.0]	31.0 [15.0, 76.0]	32.0 [15.0, 76.0]	0.408
**Race**				
Afro-Caribbean	2 (3.13%)	5 (6%)	7 (4.7%)	0.851
Asian	1 (1.56%)	1 (1.2%)	2 (1.4%)	
Hispanic/Latino	61 (95.3%)	78 (92.9%)	139 (93.9%)	
**Country of origin**				
Peru	25 (39.1%)	45 (53.6%)	70 (47.3%)	0.0682
Chile	24 (37.5%)	17 (20.2%)	41 (27.7%)	
Venezuela	7 (10.9%)	4 (4.8%)	11 (7.4%)	
Bolivia	1 (1.6%)	6 (7.1%)	7 (4.7%)	
Colombia	3 (4.7%)	3 (3.6%)	6 (4.1%)	
Haiti	1 (1.6%)	5 (6%)	6 (4.1%)	
Other	3 (4.7%)	4 (4.8%)	7 (4.7%)	
**TB disease**				
After 12-week follow-up		4 (4.8%)	4 (2.7%)	
**BCG vaccination** ^†^				
Yes	53 (88.3%)	67 (79.8%)	120 (83.3%)	0.257

TB: Tuberculosis. ^†^BCG vaccine status defined positive if BCG skin mark was present. Information was unavailable for eight participants. “Non-Mtb infected” group: samples from all individuals from the “non-TBI” groups. “Mtb infected” group: samples from all individuals with strong IGRA positive result (CD4 or CD8 IFN-γ levels ≥0.70 IU/ml) from the “pre-TBI” V2 and the “TBI” V1 groups.

### 3.3. Specific salivary IgA detection in primary analysis of groups with different stages of *M. tuberculosis* infection

**Figure 3:**
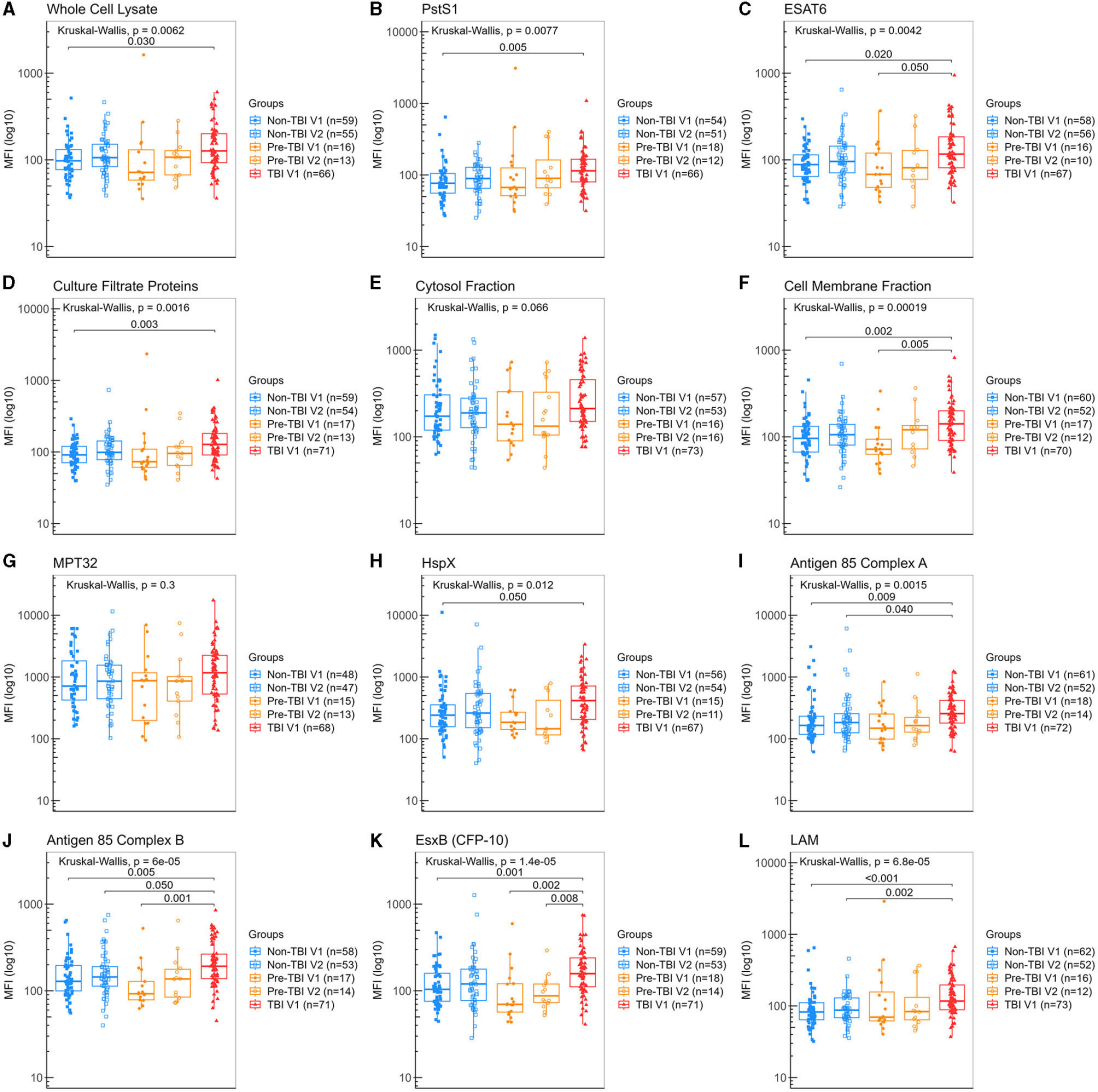
Levels of IgA against Mtb antigens in saliva samples from individuals in different stages of TB infection. Samples were incubated with bead-coupled antigens and IgA bound to bead coupled antigens was recognized with an anti-hu-IgA-R-PE secondary antibody and detected with a MAGPIX instrument (Luminex). Binding events are displayed as median fluorescence intensity (MFI) in arbitrary units (AU) and plotted in a log scale. In the box and whisker plot are represented the median with the lower and upper quantiles, and the minimum and maximum value of the data set for each group. Mtb antigens specific IgAs were as follows: **(A)** anti-whole cell lysate IgA, **(B)** anti-PstS1 IgA, **(C)** anti-ESAT-6 IgA, **(D)** anti-culture filtrate proteins IgA, **(E)** anti-cytosol fraction IgA, **(F)** anti-cell membrane fraction IgA, **(G)** anti-MPT32 IgA, **(H)** anti-HspX IgA, **(I)** anti-Ag85A IgA, **(J)** anti-Ag85B IgA, **(K)** anti-EsxB (CFP-10) IgA and **(L)** anti-LAM IgA. Groups were as follows: (1) “non-TBI” V1 (*n* = 63), (2) “non-TBI” V2 (*n* = 59), (3) “pre-TBI” V1 (*n* = 18), (4) “pre-TBI” V2 (*n* = 17) and (5) “TBI” V1 (*n* = 86). The analysis performed included only samples with ≥35 measured beads per sort; thus, each plot display the actual number of samples included in the analysis for each antigen IgA. The statistical significance was calculated using the Kruskal–Wallis test; when significant, the Mann–Whitney *U*-test with Bonferroni correction was also performed. Two-tailed *p*-values are displayed for all Kruskal–Wallis test and Bonferroni corrected *p*-values are indicated for two groups comparisons only when significant.

Detection of IgA against all Mtb antigens was found in all participant's saliva samples, including those from the “non-Mtb infected”, although at lower levels. Participants from the “TBI” V1 group had significantly higher levels of IgA anti-WCL, PstS1, CFP, HspX, Ag85A and LAM than the “non-TBI” V1 group (Figures 3A, B, D, H, I, L), with LAM exhibiting the highest difference (median 117 vs. 82.4 AU, *p* < 0.001). Moreover, participants from the “TBI” V1 group had also significantly higher levels of IgA anti-ESAT-6, CMF, Ag85B and EsxB (CFP-10) than both the “non-TBI” V1 and “pre-TBI” V1 groups (Figures 3C, F, J, K).

No differences were found between baseline (V1) and follow-up (V2) visits for all IgA specific antigens within the “non-TBI” or the “pre-TBI” group, suggesting no significant changes occurred in this short period of follow-up. The absence of rise in IgA levels from V1 to V2 in the “pre-TBI” group, suggests that the development of immunoglobulin in mucosa may take longer than the building of systemic cellular immunity as detected by an IGRA conversion, or that low antigen burden in the initial stages of TBI may not be sufficient to induce a strong humoral response in mucosa.

Additionally, the TB incidence in the country of origin (relating to previous possible environmental exposure), defined as “Low/Low-moderate” (<50 per 100, 000 inhabitants) (*n* = 37) and “Upper-moderate to Severely endemic” (≥50 per 100, 000 inhabitants) (*n* = 26) (41) did not relate with levels of specific IgA responses (Supplementary Figure 1).

### 3.4. Specific salivary IgA levels in the secondary analysis groups of TB infection

**Figure 4:**
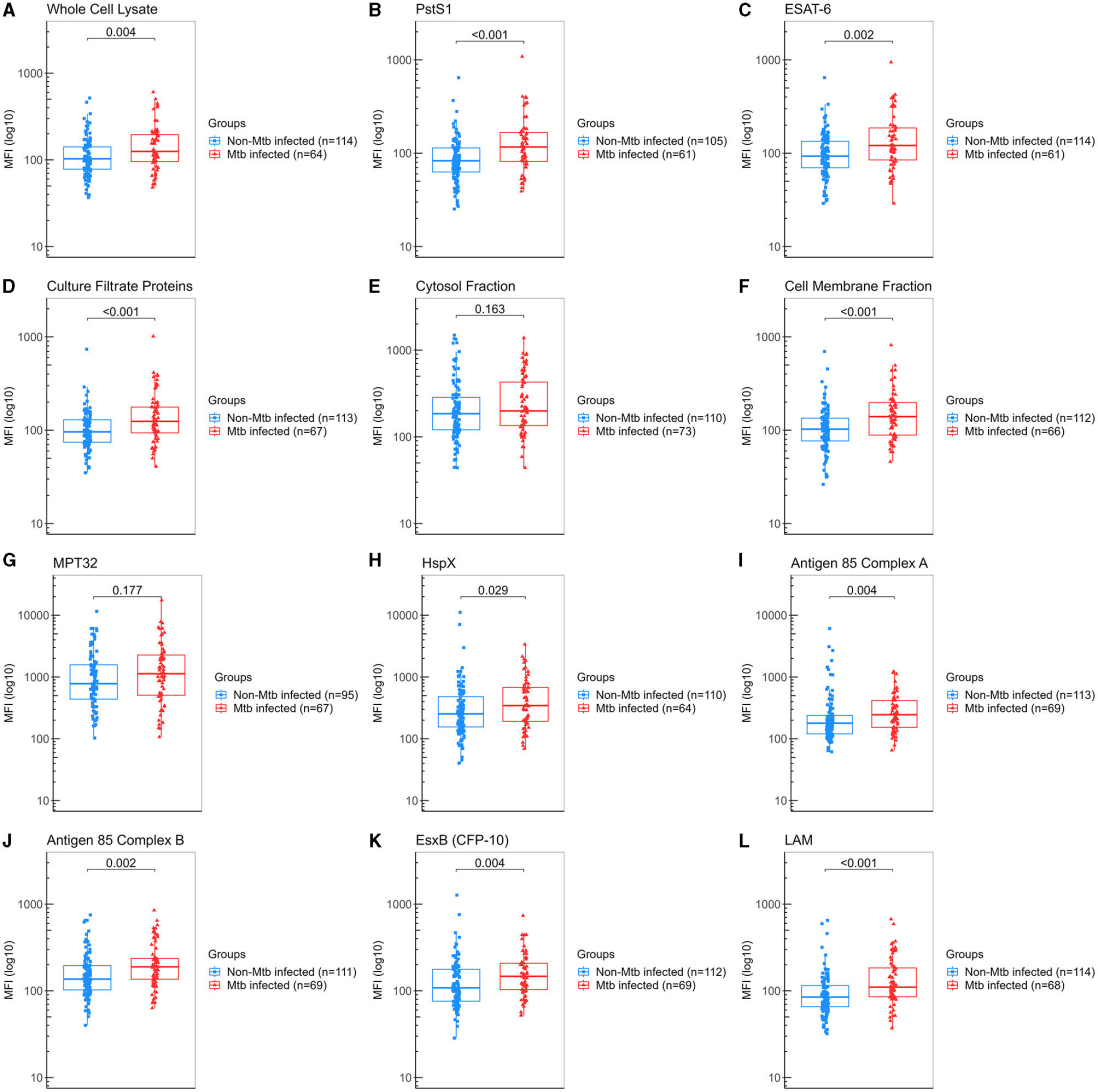
Levels of IgA against Mtb antigens in saliva samples from all “non-Mtb infected” and all “Mtb infected” samples. Samples were incubated with bead-coupled antigens and IgA bound to bead coupled antigens was recognized with an anti-hu-IgA-R-PE secondary antibody and detected with a MAGPIX instrument (Luminex). Binding events are displayed as median fluorescence intensity (MFI) in arbitrary units (AU) and plotted in a log scale. In the box and whisker plot are represented the median with the lower and upper quantiles, and the minimum and maximum value of the data set for each group. Mtb antigens specific IgAs were as follows: **(A)** anti-whole cell lysate IgA, **(B)** anti-PstS1 IgA, **(C)** anti-ESAT-6 IgA, **(D)** anti-culture filtrate proteins IgA, **(E)** anti-cytosol fraction IgA, **(F)** anti-cell membrane fraction IgA, **(G)** anti-MPT32 IgA, **(H)** anti-HspX IgA, **(I)** anti-Ag85A IgA, **(J)** anti-Ag85B IgA, **(K)** anti-EsxB (CFP-10) IgA and **(L)** anti-LAM IgA. Comparison groups were as follows: (1) “non-Mtb infected” (*n* = 122) (“non-TBI” V1 and “non-TBI” V2) vs. (2) “Mtb infected” (*n* = 86) [all contacts IGRA positive (≥0.7 IU/ml) (from the “pre-TBI” V2 and the “TBI” V1 groups)]. The analysis performed included only samples with ≥35 measured beads per sort; thus, each plot display the actual number of samples included in the analysis for each antigen IgA. The statistical significance was calculated using the Mann–Whitney *U-*test, and two-tailed *p*-values are indicated.

**Figure 5:**
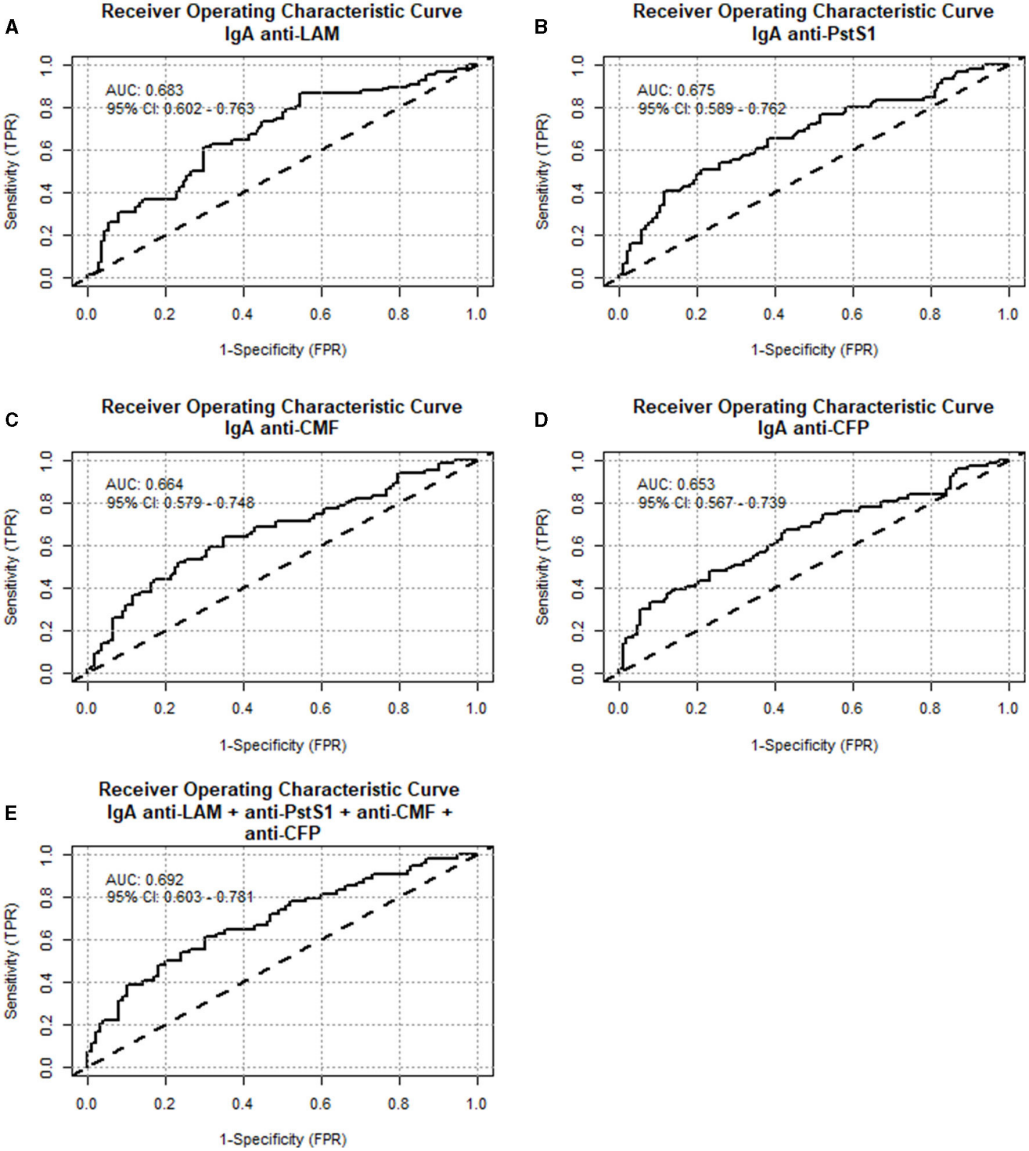
Diagnostic performance of specific IgA anti-Mtb antigens from “non-Mtb infected” and “Mtb infected” saliva samples. Receiver operating characteristic curves (ROC) were constructed to assess the discriminatory performance between “non-Mtb infected” and “Mtb infected” samples for: **(A)** anti-LAM IgA, **(B)** anti-PstS1 IgA, **(C)** anti-cell membrane fraction IgA, **(D)** anti-culture filtrate proteins IgA and **(E)** the combination of these four Mtb antigens IgA together. The area under the curve (AUC) and the confidence interval are indicated inside the plot.

A secondary analysis was performed by regrouping a total of 122 samples from “non-TBI” participants as “non-Mtb infected,” and 84 samples from the “pre-TBI” V2 and “TBI” V1 as “Mtb-infected,” as described in Section 2.1. The “Mtb-infected” group displayed significantly higher levels of IgA against WCL, PstS1, ESAT-6, CFP, CMF, HspX, Ag85A, Ag85B, EsxB (CFP-10), and LAM, compared to the “non-Mtb infected” group. The largest differences between groups were observed for anti-LAM IgA (median 110 vs. 84.8 AU, *p* < 0.001), followed by PstS1 (median 117 vs. 83 AU, *p* < 0.001), CMF (median 140 vs. 103 AU, *p* < 0.001) and CFP (median 125 vs. 96 AU, *p* < 0.001) (Figure 4). IgA anti-LAM had an AUC for TBI diagnosis of 0.683 (95% CI:0.602–0.763) with 64.71% sensitivity and 63.16% specificity at the cut-off of 94.75 AU; IgA anti-PstS1 had an AUC of 0.675 (95% CI: 0.589–0.762) with 68.85% sensitivity and 54.29% specificity at the cut-off of 87.25 AU; IgA anti-CMF had an AUC of 0.664 (95% CI: 0.579–0.748) with 68.18% sensitivity and 57.14% specificity at the cut-off of 109 AU; IgA anti-CFP had an AUC of 0.653 (95% CI: 0.567–0.739) with 67.16% sensitivity and 57.52% specificity at the cut-off of 100.5 AU (Figure 5). Combining anti-LAM, anti-PstS1, anti-CMF and anti-CFP specific IgAs did not significantly improve the discriminative ability of the model (AUC of 0.692 [95% CI: 0.603–0.781]).

Additionally, no differences were found between BCG and non-BCG vaccinated individuals from the “non-Mtb infected” and the “Mtb-infected” groups (Supplementary Figure 2).

## 4. Discussion

In the present study, we found that TBI in household contacts induces the secretion of anti-Mtb IgAs in saliva at a higher level than in the non-Mtb infected individuals, with a particularly strong IgA response against LAM, followed by PstS1, CMF and CFP. These antigens are considered Mtb-associated virulence factors, exhibit important and distinct immunomodulatory properties, and possess enzymatic activities associated with pathogenicity. LAM is a glycolipid and one of the major cell wall components accounting for up to 15% of the bacterial weight. PstS1 is a glycolipoprotein mainly located in the cell wall on the outer surface of the bacteria, acting as an immunodominant antigen in patients with TB disease, and is a major target on the antibody response in TB (42). The CMF on the other hand, contains the cytoplasmic membrane and several components of the outer lipid layer, also strongly contributing to the immune response (43). Finally, the CFP is a major repository of antigens that has been involved in the protective immune response with some of its component holding promise as tools for serodiagnosis and potential targets for tuberculosis vaccine development (44).

A considerable number of household contacts (*n* = 64, 78%) in this cohort remained uninfected at follow-up despite intense environmental exposure. Given that they also had detectable, but lower levels of Mtb-specific IgA, we cannot exclude a protective role for some of these immunoglobulins against Mtb infection acquisition. Lu et al. (45) described a similar observation in household contacts who exhibited detectable levels of IgA, IgG and IgM in plasma against several Mtb antigens, despite having a negative IGRA or TST (45).The presence of an existing humoral immune response could possible relate to the low percentage of individuals (around one third) that acquire a TBI after exposure. In accordance with this hypothesis, we observed a non-significant trend toward higher levels of IgA anti-Ag85B and anti-EsxB (CFP-10) at the baseline visit for the “non-TBI” participants than for the individuals from the “pre-TBI” group, suggesting a possible protective role for these mucosal antibodies. Although a larger sample could delucidate this hypothesis, these results are in line with a plasma proteome array study that included 4000 Mtb antigens showing that at baseline and after 3 months of exposure, individuals who remained uninfected exhibited higher levels of IgG against certain antigens than those who acquired a TBI, suggesting that the humoral immune response might have a role in infection prevention (46). Also supporting the notion that humoral immunity can provide a natural protection against Mtb, Fletcher et al. (47) recently showed that the presence of anti-Ag85A IgG in serum was associated with a lower risk of developing TB disease in the following 3 years in infants BCG-vaccinated at birth (47). In addition, the intraperitoneal injection of total IgG from plasma samples from health care workers with antibodies against Mtb surface antigens offered moderate protection against Mtb infection in an aerosol mouse challenge model (48). Nonetheless, the protective role of immunoglobulins seems to be dependent and critical upon their isotype (49). While specific IgA and IgG antibody responses against LAM and HBHA are developed by health care workers exposed to Mtb, a functional difference in Mtb inhibitory activity has been described: IgA antibodies inhibit infection and reduce intracellular bacterial load, whereas IgG antibodies can promote mycobacterial invasion *in vitro* (49).

Interestingly, the protective role of mucosal IgA has been further explored in vaccine development studies. Mice and non-human primates delivered with pulmonary mucosal BCG develop a reduced local pathology and robust protection against Mtb challenge, whereas standard intradermal injection fails (50, 51). Moreover, vaccination by endobronchial instillation prevents Mtb infection in rhesus macaques and an increase in IgA correlates with a local protective immunity (52). Mucosal delivery of BCG vaccine has also shown to provide enhanced protection over systemic BCG by inducing T cell immunity in the airways and lung tissue in a mouse model, supporting the hypothesis that mimicking the route of infection can potentiate vaccine efficacy (53).

Another relevant finding of this study is that all participants, irrespective of TBI status, had detectable levels of salivary IgA against all Mtb antigens evaluated, with levels varying depending on the one targeted. These results may relate to a pre-existing mucosal immune response due to previous—unrecognized—Mtb exposure, previous BCG vaccination, past exposure to other environmental mycobacteria (54) or to a possible cross-reactivity with other oral microbial species (55–57). Genetic diversity among Mtb strains (33) may have also had an impact on antibodies' conformation. Host genetic differences in humoral immune response, and antibody relative immunogenicity and affinity, may all have an impact on IgA expression (58). Additionally, person-to-person heterogeneity of antigen recognition (33–35, 59) can also led to intrinsic high variation in antibodies' specificity.

The use of saliva is valuable as a non-invasive and easy to obtain clinical sample, with known promise in TB diagnosis (60–63). However, its nature carries intrinsic difficulties due to large variability in its composition and viscosity, as well as differences in the flow rate related to age, time of day, and health status, among others (64–66). Saliva is a complex matrix with high levels of IgA and the presence of aggregates.

As study limitations, for the assay validation, we did not have internationally validated positive and negative controls, therefore we had to rely on saliva samples from patients with TB disease and individuals with presumed negative exposure. Additionally, due to the nature of saliva, we cannot rule out non-specific bindings from saliva components and cross-reactivity from BCG vaccination or past exposure to other environmental mycobacteria. Furthermore, the sample size of individuals with a newly acquired TBI was small, as most of the cohort participants were already infected at baseline assessment.

In conclusion, this study is the first to analyze specific salivary IgA against multiple Mtb antigens in a cohort of household contacts with TBI, including individuals with prospective follow-up (pre and post-TBI acquisition). Our results show that anti-Mtb specific salivary IgA are detectable and vary significantly in different stages of TB infection, with highest levels obtained in individuals with established TBI and lower levels obtained in the “non-Mtb infected,” and in those exposed that later progressed into a new infection. These findings strongly warrant further exploration of mucosal humoral immunity response in other stages of TB infection to assess their kinetics, as well as in other respiratory infections, to explore its role in TB acquisition risk, progression or treatment effectiveness marker.

## Data availability statement

The original contributions presented in the study are included in the article/Supplementary material, further inquiries can be directed to the corresponding author.

## Ethics statement

The studies involving humans were approved by the Institutional Review Board from the Pontificia Universidad Católica de Chile. The studies were conducted in accordance with the local legislation and institutional requirements. The participants provided their written informed consent to participate in this study.

## Author contributions

CR-T: Formal analysis, Investigation, Methodology, Writing—original draft, Writing—review and editing. RN: Investigation, Methodology, Resources, Writing—review and editing. PG: Writing—review and editing. AG: Methodology, Writing—review and editing. NS-M: Methodology, Writing—review and editing. MB: Conceptualization, Funding acquisition, Methodology, Writing—review and editing, Investigation, Resources, Validation, Writing—original draft.
