# Supplementary material for: Differential levels of anti-Mycobacterium tuberculosis-specific IgAs in saliva of household contacts with latent tuberculosis infection
Source: Front Med (Lausanne). 2023 Oct 6;10:1267670. doi: 10.3389/fmed.2023.1267670 (PMC10587581; doi:10.3389/fmed.2023.1267670)
Supplement: Supplementary file 1 [file Data_Sheet_1.docx]

**Supplementary Material**

**Differential levels of anti-*Mycobacterium tuberculosis*-specific IgAs in saliva of household contacts with latent tuberculosis infection.**

**Cinthya Ruiz-Tagle^1^, Rodrigo Naves^2^, Patricia García^3^, Anna Günther^4^, Nicole Schneiderhan-Marra^4^ and María Elvira Balcells^1^***

* **Correspondence:** María Elvira Balcells: ebalcells@uc.cl

Legends Suppl. Figures 1-2

Suppl. text, Appendix 1

Supplementary Figure 1. Levels of IgA against Mtb antigens in saliva samples from individuals of the “non-TBI” V1 group classified by TB incidence of their country of origin. Classification was based on the TB incidence of their country of origin as follows: “Low/Low-moderate” (<50 per 100,000 inhabitants) and “Upper-moderate to Severely endemic” (≥50 per 100,000 inhabitants). Binding events are displayed as median fluorescence intensity (MFI) in arbitrary units (AU) and plotted in a log scale. In the box and whisker plot are represented the median with the lower and upper quantiles, and the minimum and maximum value of the data set for each group. Mtb antigens specific IgAs were as follows: (A) anti-whole cell lysate IgA, (B) anti-PstS1 IgA, (C) anti-ESAT-6 IgA, (D) anti-culture filtrate proteins IgA, (E) anti-cytosol fraction IgA, (F) anti-cell membrane fraction IgA, (G) anti-MPT32 IgA, (H) anti-HspX IgA, (I) anti-Ag85A IgA, (J) anti-Ag85B IgA, (K) anti-EsxB (CFP-10) IgA and (L) anti-LAM IgA. Groups were as follows: “Low/Low-moderate” (<50) (n=37) and “Upper-moderate to Severely endemic” (≥50) (n=26). The analysis performed included only samples with ≥35 measured beads per sort; thus, each plot display the actual number of samples included in the analysis for each antigen IgA. The statistical significance was calculated using the Mann-Whitney U test, and two-tailed p-values are indicated.

Supplementary Figure 2. Levels of IgA against Mtb antigens in saliva samples from BCG and non-BCG vaccinated individuals from the “non-Mtb infected” and “Mtb-infected” groups. Binding events are displayed as median fluorescence intensity (MFI) in arbitrary units (AU) and plotted in a log scale. In the box and whisker plot are represented the median with the lower and upper quantiles, and the minimum and maximum value of the data set for each group. Mtb antigens specific IgAs were as follows: (A) anti-whole cell lysate IgA, (B) anti-PstS1 IgA, (C) anti-ESAT-6 IgA, (D) anti-culture filtrate proteins IgA, (E) anti-cytosol fraction IgA, (F) anti-cell membrane fraction IgA, (G) anti-MPT32 IgA, (H) anti-HspX IgA, (I) anti-Ag85A IgA, (J) anti-Ag85B IgA, (K) anti-EsxB (CFP-10) IgA and (L) anti-LAM IgA. Groups were as follows: 1) “Non-Mtb infected/BCG” (n=101), 2) “Non-Mtb infected/non-BCG” (n=13), 3) “Mtb infected/BCG” (n=67), 4) “Mtb infected/non-BCG” (n=17). The analysis performed included only samples with ≥35 measured beads per sort; thus, each plot display the actual number of samples included in the analysis for each antigen IgA. The statistical significance was calculated using the Mann-Whitney U test, and two-tailed p-values are indicated.

**Supplementary text**

**Appendix 1. Multiplex bead-based Luminex immunoassay**

***Patient selection***

For assays development, we collected negative and positive saliva samples based on the following criteria.

*Negative samples from “non-TB exposed” controls*: Asymptomatic healthy volunteers, >18 years old and with recent (<3 months) negative interferon-γ release assay test. Volunteers with history of previous TB, contact with people with TB, use of any immunosuppressor, use of inhaled corticosteroids, known immunosuppression, autoimmune diseases, known HIV infection, current use of inhaled drugs, current use of antibiotics (<1 month), current pregnancy, and migrants from high TB incidence country were excluded.

*Positive samples from patients with PTB disease*: Individuals >18 years with pulmonary TB under successful treatment for at least 60 days. Patients with any immunosuppression or immunosuppressant use, use of inhaled corticosteroids, autoimmune diseases, known HIV infection, inhaled drugs, current pregnancy, and drug-resistant TB were excluded.

All eligible participants provided written informed consent, according to institutional requirements.

***Sampling procedures***

Participants were instructed to pool the saliva inside their closed mouth for as long as they could and to deposit it inside a sterile container. The process, repeated until they collected about 1-4 ml, took approximately 20 minutes depending on the participant. After collection, samples were kept on ice until centrifuged at 10,000 x g for 15 min at 4°C, aliquoted and stored at -80°C until measurements.

Blood: Blood samples were drawn from “non-TB” exposed controls to assess TBI status with a standard IGRA test (QuantiFERON®-TB Gold Plus (QFT), QIAGEN, Hilden, Germany). Only samples from participants with a negative IGRA were included for assay development.

***Antibodies***

The following reagents were obtained through BEI Resources, NIAID, NIH: polyclonal anti-*Mycobacterium tuberculosis* Whole Cell Lysate (antiserum, Rabbit), NR-13819; polyclonal anti-*Mycobacterium tuberculosis* Whole Cell Lysate minus LAM (antiserum, rabbit), NR-13820; polyclonal anti-*Mycobacterium tuberculosis* Cytosol minus LAM (antiserum, rabbit), NR-13802; polyclonal anti-*Mycobacterium tuberculosis* CFP (antiserum, rabbit), NR-13808; polyclonal anti-*Mycobacterium tuberculosis* CFP minus LAM (antiserum, rabbit), NR-13809; polyclonal anti-*Mycobacterium tuberculosis*, Cell Wall (CW) (antiserum, rabbit), NR-44075; polyclonal anti-*Mycobacterium tuberculosis* ESAT6 (Gene Rv3875) (antiserum, rabbit), NR-13803; monoclonal anti-*Mycobacterium tuberculosis* PhoS1/PstS1 (Gene Rv0934), Clone IT-15 (TB72) (produced in vitro), NR-13605; monoclonal anti-*Mycobacterium tuberculosis* PhoS1/PstS1 (Gene Rv0934), Clone IT-21 (HYT28) (produced in vitro), NR-13608; monoclonal anti-*Mycobacterium tuberculosis* PhoS1/PstS1 (Gene Rv0934), Clone IT-47 (HBT12) (produced in vitro), NR-13652; monoclonal anti-*Mycobacterium tuberculosis* PhoS1/PstS1 (Rv0934), Clone IT-23 (TB71) (produced in vitro), NR-13790; polyclonal anti-*Mycobacterium tuberculosis* LAM (antiserum, rabbit), NR-13821, and monoclonal anti-*Mycobacterium tuberculosis* LAM, Clone CS-35 (produced in vitro), NR-13811. Penta-His antibody, monomeric human IgA, human IgA lambda (dimer), goat anti-human IgA and goat anti-human IgA-RPE were purchased from QIAGEN (#34660, Hilden, Germany), Abcam (ab91025, Cambridge, United Kingdom), Gentaur GmbH (P 444, Aachen, Germany) and Jackson ImmunoResearch Inc. (109-005-011 and 109-115-011, Pennsylvania, United States), respectively.

***Development of a multiplex bead-based Luminex immunoassay***

The selected Mtb antigens (n=12) were coupled to magnetic color-coded beads at two different concentrations (5 µgx10^6^ beads or 10 µgx10^6^ beads) using ECD/Sulfo-NHS chemistry according previous publication [1] with some modifications. The antigen-coupling efficiency was confirmed using serial dilutions of the anti-antigen specific antibody or an anti-His tag antibody for the recombinant proteins. For whole cell lysate, ESAT-6, PstS1, LAM and culture filtrate proteins we performed serial dilutions (100, 200, 400, 800, 1600, 3200 and 6400) of antibodies rbt-a-WCL (NR-13819), rbt-a-WCL (w/o LAM) (NR-13820), rbt-a-ESAT-6 (NR-13803), ms-a-PstS1 (NR-13605), ms-a-PstS1 (NR-13608), ms-a-PstS1 (NR-13652), ms-a-PstS1 (NR-13790), rbt-a-LAM (NR-13821), ms-a-LAM (NR-13811), ms-a-LAM (NR-13812), rbt-a-CFP (NR-13808) and rbt-a-CFP (w/o LAM) (NR-13809), respectively. For cytosol fraction we performed a serial dilution (400, 800, 1600, 3200, 6400, 12800 and 25600) of rbt-a-Cytosol (w/o LAM) (NR-13802) and for cell membrane fraction, a serial dilution (50, 100, 200, 400, 800, 1600 and 3200) of rbt-a-Cell Wall (NR-44075). Finally, for MPT32, HspX, Ag85A, Ag85B and EsxB (CFP-10) we performed serial dilutions of 2500, 1250, 625, 313, 156 and 78.1 ng/ml using the anti-His tag antibody. The corresponding antigen**-**antibody binding was detected with a secondary phycoerythrin (PE)-labeled antibody (7.5 µg/ml). The results showed that coupled antigens exhibited similar kinetics and median fluorescence intensity (MFI) with both concentration of antigens. Moreover, the results indicated that the coupling was successful for all antigens although recombinant HspX, Ag85A and EsxB exhibited lower MFI due to steric hindrance or lower access. To assess if the coupled Mtb antigens could be detected by specific mucosal IgA, first we tested several buffer combinations with different salt and detergent composition to reduce nonspecific interactions and matrix effect using control beads coupled with human-IgA and human dimeric IgA. The best buffer combination was then used to test saliva samples from healthy individuals (n=9) and patients with active TB (n=6) at three dilutions (1:4, 1:8 and 1:16). Results showed that all coupled Mtb antigens bound specific IgA and that most samples displayed a higher MFI with the lowest dilution (1:4), even though contrary results or no change was observed for a few samples probably due to interfering material that could not be mitigated even at higher dilutions. Differences between groups was not assess do to the low number of samples.

***Assay validation***

The assay was standardized and validated it according to the assay validation guidelines from Jacobson, the Food and Drug Association (FDA) and the European Medicines Agency (EMA) [2–4]. Intra-assay coefficients of variation (CVs) were calculated per antigen based on the measurement of twenty-one replicates of the same sample (saliva samples from 4 patients with TB disease). We observed intra-assay CVs <20% for all antigens (12/12). For inter-assay variation the experiment was repeated three times by running triplicates of each. The inter-assay CV were <20% for almost all antigens in all the samples. Specifically, the inter-assay CVs were <20% for all samples (4/4) for 1 antigen (1/12), for 3 samples for 9 antigens and for 2 samples for 2 antigens. The stability performance was calculated considering the fresh sample as reference. The one regarding three freeze/thaw cycles showed a sample recovery between 80 and 120% for most of the antigens. The sample from one patient with TB disease had a higher recovery rate for the second freeze/thaw cycle for the antigens whole cell lysate, ESAT-6 and cytosol fraction, and for the first and second freeze/thaw cycles for EsxB. Also, the sample from one “non-TB exposed” control had a higher recovery percentage for the third freeze/thaw cycle for MPT32, HspX and Ag85A. The stability performance of the samples regarding the storage conditions at room temperature (RT) and 4°C for 2, 4 and 24 hours showed that the sample from one “non-TB exposed” control had a poor recovery percentage (below 80%) for nine antigens for all storage conditions. This sample also had a low recovery rate for the storage conditions at 4°C for 24 h, for all storage conditions at RT for the cytosol fraction and Ag85B, and at RT for 4 h and 24 h for Ag85A. Moreover, for the condition at RT for 24 h the bead count was lower than 35 beads, so MFI was not registered for analysis. On the other hand, the sample from one patient with TB disease had a recovery rate above 80% for five antigens for all storage conditions, and for six antigens at 4°C and RT for 2 and 4 h. Finally, for the dilution linearity, four samples were evaluated and diluted (dilution factor: 1:2, 1:4, 1:8 and 1:16) to assess the percentage of recovery calculated considering as reference the sum of the average MFI multiplied by the dilution factor (MFIxDF) of each dilution. For all antigens, a good percentage of IgA recovery was obtained for dilution 1:4 for almost all samples.

Overall, saliva samples were stable after repeated freeze/thaw cycles and in some cases freeze/thaw cycles increased the MFI, probably due to precipitation by centrifugation of unwanted/interfering material. Also, samples can be stored at 4°C or RT for short periods of time without compromising the sample or the assay.

**References**

1. Broger T, Roy RB, Filomena A, et al. Diagnostic performance of tuberculosis-specific IgG antibody profiles in patients with presumptive tuberculosis from two continents. Clin Infect Dis. **2017**; 64(7):947–955.

2. Jacobson RH. Validation of serological assays for diagnosis of infectious diseases. OIE Rev Sci Tech. **1998**; 17(2):469–486.

3. US Food and Drug Administration. Guidance for industry: bioanalytical method validation. **2001**; (May).

4. Agency EM. Guideline on bioanalytical method validation. **2009**; EMEA/CHMP/EWP/192217/2009.
