# Supplementary figures and images for: Differential levels of anti-Mycobacterium tuberculosis-specific IgAs in saliva of household contacts with latent tuberculosis infection
Source: Front Med (Lausanne). 2023 Oct 6;10:1267670. doi: 10.3389/fmed.2023.1267670 (PMC10587581; doi:10.3389/fmed.2023.1267670)

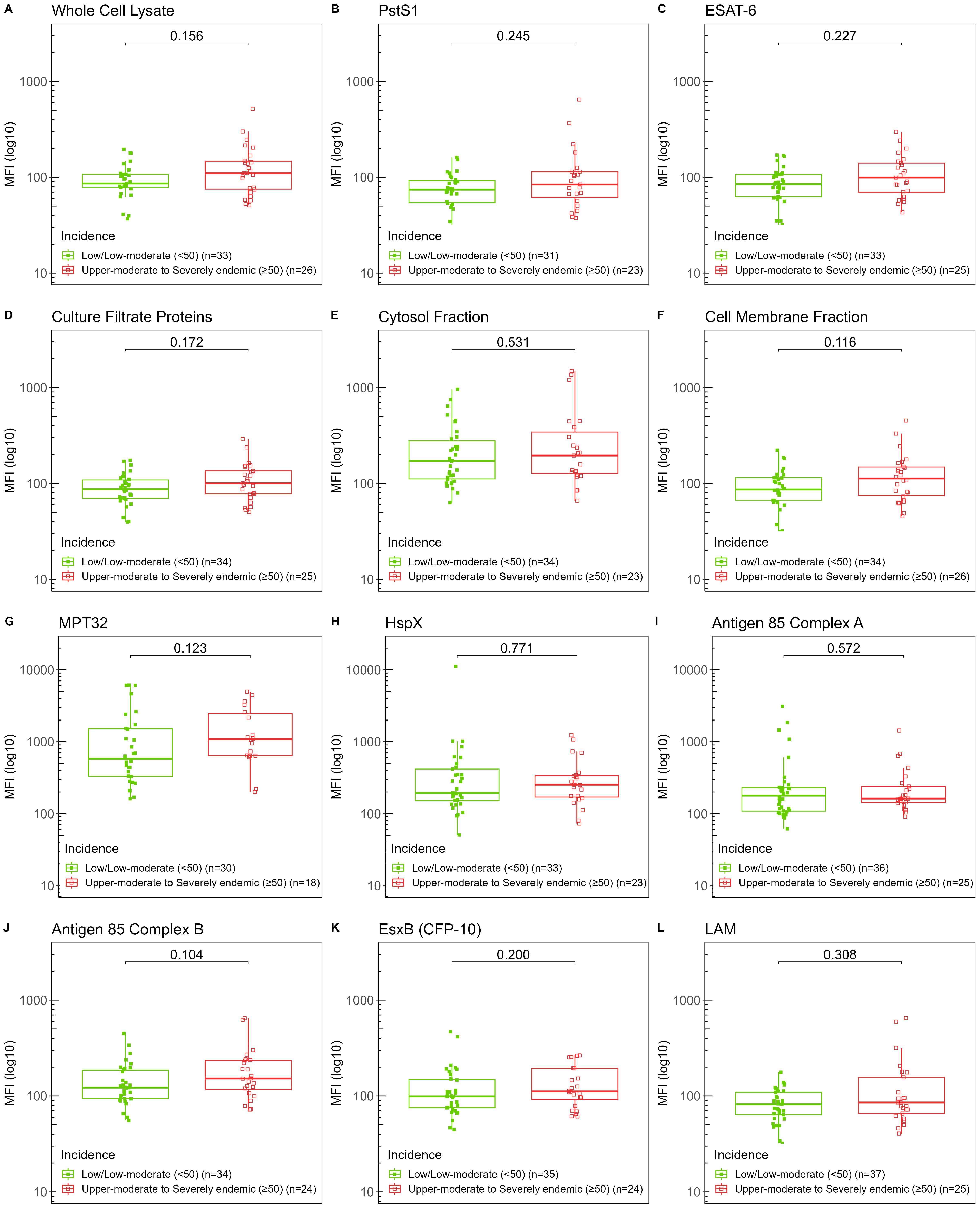

Supplement: Supplementary file 2 [file Image_1.tiff]

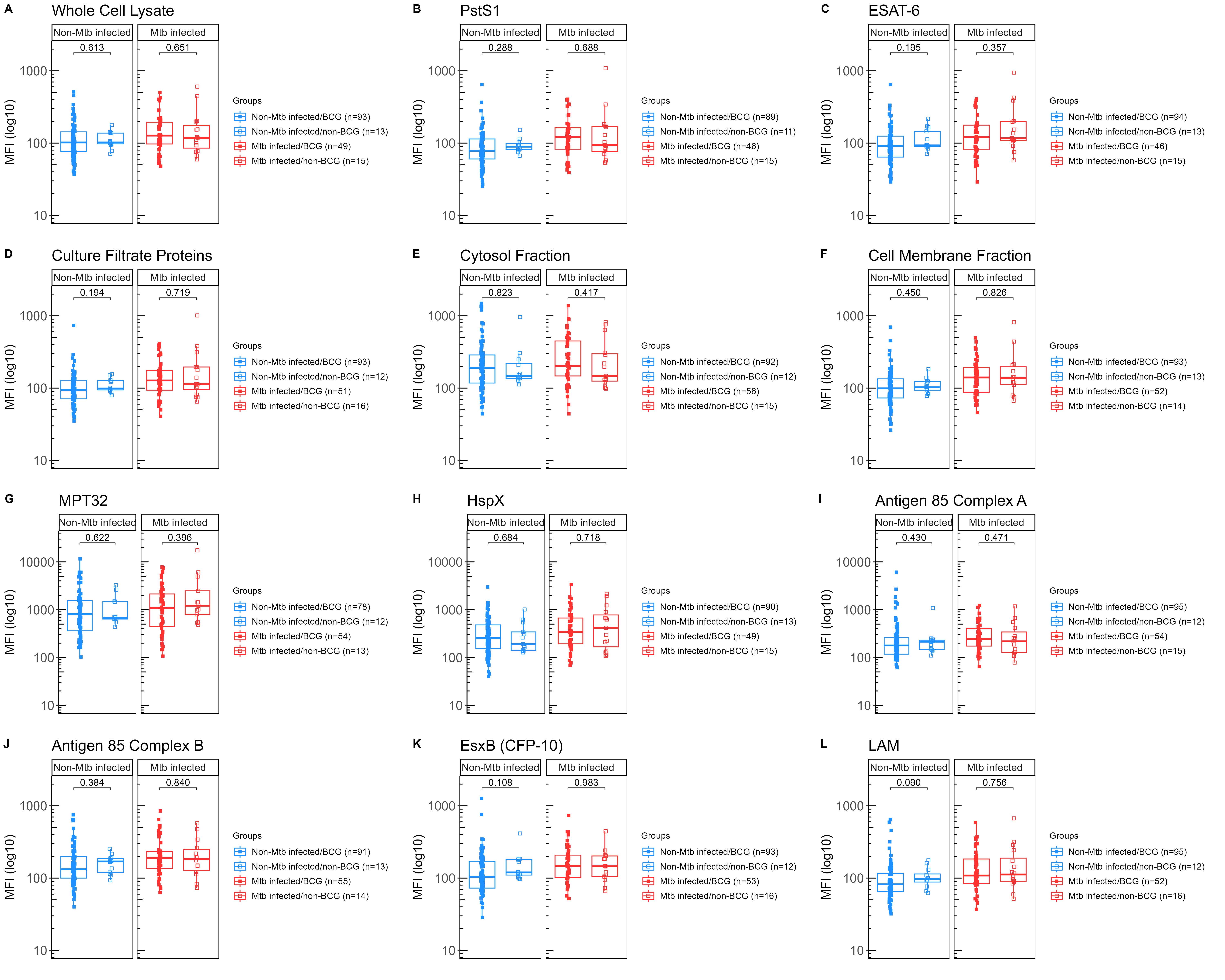

Supplement: Supplementary file 3 [file Image_2.TIFF]
